# Residue of Paclobutrazol and Its Regulatory Effects on the Secondary Metabolites of *Ophiopogon japonicas*

**DOI:** 10.3390/molecules24193504

**Published:** 2019-09-27

**Authors:** Lixia Zhang, Zuliang Luo, Shengrong Cui, Lei Xie, Jing Yu, Deying Tang, Xiaojun Ma, Yan Mou

**Affiliations:** 1 Institute of Medicinal Plant Development, Chinese Academy of Medical Sciences & Peking Union Medical College, Beijing 100193, China; 13988194288@163.com (L.Z.); zuliangluo@163.com (Z.L.); c1061729635@163.com (S.C.); xielei1996@163.com (L.X.); 2 Yunnan Branch, Institute of Medicinal Plant Development, Chinese Academy of Medical Sciences, Jing Hong 666100, China; yujingynu1113@163.com (J.Y.); tdy629@126.com (D.T.)

**Keywords:** *Ophiopogon japonicus*, paclobutrazol, residue, steroidal saponins, flavonoids

## Abstract

Currently, paclobutrazol is excessively used in the planting process of *Ophiopogon japonicus* (*O. japonicus*) due to its important role in regulating the growth of tuber roots, ultimately increasing the yield and shortening the growth cycle of Ophiopogonis Radix. For insight into this process and the potential risks of paclobutrazol and its mediated consequences on the secondary metabolites in Ophiopogonis Radix, corresponding high performance liquid chromatography-tandem mass spectrometric methods (HPLC-MS/MS) were developed in this study and then applied to Ophiopogonis Radix, soil, and water samples. The results demonstrated the detection of different levels of paclobutrazol residue were in Ophiopogonis Radix, soil, and water samples. In addition, the quantitative results of the secondary metabolites showed that paclobutrazol significantly decreased four steroidal saponins in Ophiopogonis Radix, especially ophiopogonin D, where the content was decreased from 824.87 to 172.50 mg/kg. Concurrently, ophiopogonanone C, a flavonoid in Ophiopogonis Radix, also significantly decreased from 2.66 to 1.33 mg/kg. In conclusion, the residual paclobutrazol and its negative regulation on the secondary metabolism of Ophiopogonis Radix brings potential hazards to the environment and human health. These results provide more comprehensive data that can be used for the reassessment of the use of paclobutrazol in *O. japonicus* and the formulation of related standards.

## 1. Introduction

*Ophiopogon japonicus* (*O. japonicas*) (L.f.) Ker-Gawl is an evergreen perennial herb in the Liliaceae family. At present, it is planted primarily in Santai County of Sichuan Province, China. Ophiopogonis Radix, the dried *O. japonicus* tuberous root, is known to be an important traditional Chinese herbal medicine and functional food [1,2,3]. Ophiopogonis Radix is recorded in various versions of Chinese Pharmacopoeia and is used to treat hot dry lung cough, yin deficiency pains cough, sore throat and pharynx, internal heat dispersion thirst, vexation, insomnia, and constipation [4]. In 2002, Ophiopogonis Radix was approved for use as a functional food ingredient by the Ministry of Health of the People’s Republic of China [5]. To date, the China Food and Drug Administration (CFDA) has approved patented drugs, namely, the ShenMai injection/granule and the XuanMai Gan Jie capsule/granule, which contain Ophiopogonis Radix as the main medicinal ingredient [6]. In addition, it is used as a yin-nourishing remedy for the treatment of cardiovascular disease and acute and chronic inflammation in the clinic and has been included in a variety of functional food supplements aimed at promoting health and longevity, such as nutrition health teas [7]. Steroidal saponins, flavonoids, and polysaccharides are the main active ingredients of Ophiopogonis Radix. To date, more than 30 flavonoids, 70 saponins, and 10 bioactive polysaccharides have been isolated from Ophiopogonis Radix [1,8]. Modern pharmacological studies show that Ophiopogonis Radix is rich in flavonoids, steroidal saponins, and polysaccharides, which have beneficial effects on immunomodulatory, cardioprotective, neuroprotective, antimicrobial, antihyperlipidaemic, antioxidant, anticancer, anti-inflammation, and antidiabetic activities [1,9].

With the ever-increasing use of herbal medicines and the rapid expansion of their market, most of the wild resources of medicinal plants have dried up. Hence, most of the commonly used herbal medicines, such as Ophiopogonis Radix, are now obtained by artificial planting. *O. japonicus* usually requires three years from planting to harvest, according to the history of planting [9]. However, in recent years, with the demand of the market and the maximization of farmers’ economic interests, a cultivation mode of one-year-old *O. japonicus* has been developed. At the same time, chemical fertilizers, pesticides, and plant regulators are widely used in the planting process. Paclobutrazol, a plant growth regulator (PGR), has been widely used in the cultivation of *O. japonicas* to regulate the growth of tuber roots and ultimately increase the yield and shorten the planting cycle of *O. japonicus*.

According to their mode of action, PGRs can be divided into growth promoters, growth retardants, and growth inhibitors. Paclobutrazol is a growth retardant; it has the function of delaying plant growth, inhibiting stem elongation, shortening internodes, promoting branching and tillering, promoting the formation of the root system, increasing plant stress resistance, and increasing the yield [10]. Owing to its specific properties, paclobutrazol is widely used in the cultivation of medicinal plants, especially for root herbs [11]. In the early stage of investigation on the genuine producing areas of Ophiopogonis Radix (Santai, Sichuan, China), we found that paclobutrazol is widely used in *O. japonicus* planting. The amount of paclobutrazol suspension concentrate (25%) used per 667 m^2^ is about 3–5 kg. In addition, up to 40 batches of Ophiopogonis Radix collected from production areas and traditional Chinese medicine (TCM) markets in China were analyzed, and the detection rate of paclobutrazol in Ophiopogonis Radix was 100%. Moreover, the residual level of paclobutrazol in some Ophiopogonis Radix samples exceeded the recommended maximum residue limit (MRL), according to GB 2763-2016 [12]. Before that, Zhao et al. also found the similar results [13].

Currently, indiscriminate and excessive use of PGR has affected the quality of Chinese medicinal materials and is threatening public health. In addition, an inherent impact associated with the use of some PGRs is long-lasting residual activity in agricultural soil, which seriously inhibits subsequent crop growth [14]. Moreover, a number of countries and international organizations have enacted regulations by setting up the MRL of PGR in various crops, fruits, and vegetables [15,16,17,18]. However, there is no relevant regulation for PGR in TCM in China and other countries. In recent years, Chinese researchers have successively studied the effect of paclobutrazol on the yield and quality of *O. japonicus*, the residue of paclobutrazol in Ophiopogonis Radix, and soil from its production area. They found that the total saponin, total flavonoid, and total polysaccharide contents in Ophiopogonis Radix are distinctly reduced after the spraying of paclobutrazol [19,20,21]. However, the available information is limited, and it is necessary to carry out further systematic and in-depth studies to evaluate the effect of paclobutrazol on *O. japonicus*.

Because of the complexity of *O. japonicus* polysaccharides, the total polysaccharide content is mainly determined by the phenol-sulfuric acid colorimetric method at present. However, for the quantitative analysis of flavonoids and saponins, a variety of analytical methods such as liquid chromatography-diode array detection (LC-DAD), liquid chromatography-evaporating light scattering detection (LC-ELSD), and liquid chromatography-tandem mass spectrometry (LC-MS/MS) have been developed. Although these methods have contributed significantly to the quality control of Ophiopogonis Radix, they have their own drawbacks in terms of rapidly, sensitively, and selectively measuring the targeted analytes.

Therefore, this study collected samples from the paclobutrazol-treated experiment. Then, we attempted to develop a practical strategy based on the LC-MS/MS-targeted technique by employing an information-dependent acquisition (IDA) approach by multiple reaction monitoring (MRM) as the survey scan and the enhanced product ion (EPI) as the dependent scan for screening, identifying, and quantifying the residues of paclobutrazol in Ophiopogonis Radix, soil, and water samples. In addition, a sensitive and rapid LC-MS/MS method was developed for the simultaneous determination of nine secondary metabolites in *O. japonicus*, including two types of bioactive components (four steroidal saponins and five flavonoids). Finally, the advantages and disadvantages of paclobutrazol were evaluated according to these experimental results.

## 2. Results and Discussion

### 2.1. HPLC-MS/MS Conditions

In order to find the optimum MS behaviors and electrospray ionization (ESI) source parameters for the identification and quantification of the targeted analytes, preliminary experiments were carried out. A standard solution (100 ng/mL) of each analyte was directly introduced into the mass spectrometer by a syringe pump (Harvard apparatus, South Natick, MA, USA) at a flow rate of 10 μL/min. The mass spectra of positive and negative ion modes were compared under full scan mode to choose the optimum ionization mode and identify the parent ions of the targeted analytes. At the same time, the important MS parameter declustering potential (DP) was optimized to obtain the maximum sensitivity [22]. With the selection of precursor ions, product ion scanning and MRM scanning were conducted to obtain specific fragment ions and the collision energies (CEs) in favor of highest response and least interference. One precursor ion coupled with two characteristic product ions for each analyte was optimized. The product ion with the highest signal-to-noise (S/N) ratio and intensity was chosen for quantification. The optimized MRM transitions and the corresponding parameters are shown in Table 1.

To get synchronous supplementary confirmation of paclobutrazol residue, an IDA experiment was employed to automatically trigger EPI scans by analyzing MRM signals. The IDA criteria included selecting the two most intense precursor ions after dynamic background subtraction of the survey scan, and the former target ions were never excluded. The mass tolerance for precursor ions was 250 mDa. EPI spectra were acquired using intensities exceeding 1000 counts per second (cps) over a mass range of *m*/*z* 50–350 for product ions at a scan rate of 10,000 Da/s. The CE was set at 30 V, and the collision-activated dissociation (CAD) was high.

**Figure 1:**
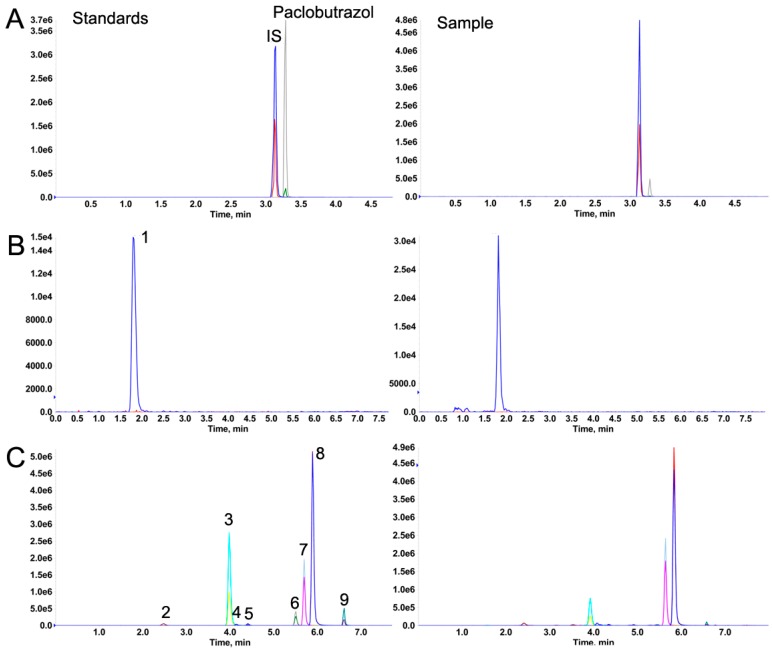
Multiple reaction monitoring (MRM) chromatograms of the mixture standards of forchlorfenuron (IS) and paclobutrazol (**A**), the secondary metabolites of Ophiopogonis Radix, under positive (**B**) and negative (**C**) ion modes: SRa (1), SC (2), FE (3), SD (4), SD’ (5), FA (6), FNA (7), FNB (8), and FC (9). The left column shows the standards and the right column shows the samples.

The composition of the mobile phase can strongly influence the performance of the ionization process and the separation of the analytes. To provide the best resolution and peak-shape response intensity, we finally determined the optimum mobile phase composition and chromatographic conditions by referring to previous studies [7,23]. The optimum chromatographic conditions are described in the Materials and Methods section. The typical MRM chromatograms for the targeted analytes under the optimized MS and chromatographic conditions are shown in Figure 1.

### 2.2. Sample Pretreatment

**Figure 2:**
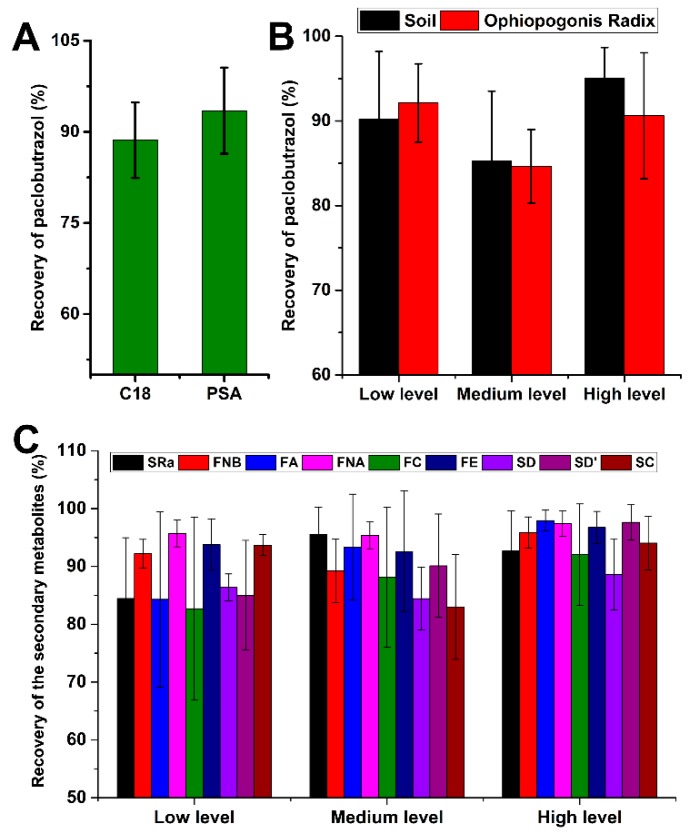
Effects of different sorbents on the recovery of paclobutrazol (**A**); the extraction recovery of paclobutrazol from soil and Ophiopogonis Radix (**B**); the extraction recovery of the secondary metabolites from Ophiopogonis Radix (**C**).

For accurate and effective detection of the targeted analytes in complex matrices, the ultrasound-assisted solid–liquid extraction method is a convenient, quick, and cost-effective approach to the extraction of the analytes. It has become the preferred method for sample extraction in rapid analyses. Based on the solvents used for pesticide extraction in previous research [24,25], acetonitrile containing 1.0% formic acid was selected as the extraction solvent. Due to the complex matrix of the *O. japonicus* samples, interferents were present in the extraction solution. These interferents had the potential to enhance or inhibit the ion signals of the target compounds and result in contamination of the ion source. Hence, a clean-up was the performed step by QuEChERS to remove the matrix interference. To evaluate the capacity for removing impurities in matrix, sorbents based on primary secondary amine (PSA) and octadecylsilyl (C_18_) were investigated separately in a preliminary analysis. The amount of all sorbents (PSA and C_18_) used in the comparison was 50 mg, which was added to 150 mg anhydrous MgSO_4_. As shown in Figure 2A, with PSA as the clean-up sorbent, paclobutrazol showed a higher recovery level than C_18_. Finally, 10 mL of acetonitrile containing 1.0% formic acid was used for extraction, and the composition of 50 mg PSA and 150 mg anh. MgSO_4_ was chosen for clean-up. The recovery rates at the three spiking levels of paclobutrazol, 20, 50, and 100 ng/L, were within the acceptable range (84.6%–95.1%, Figure 2B).

To obtain the optimum extraction conditions of the secondary metabolites, the related extraction conditions of the extraction solvent (methanol, ethanol, and acetonitrile), concentration (50%, 70%, and 100%, *v*/*v*), extraction volume (10, 20, and 30 mL), and extraction time (15 min, 30 min, 45 min, and 1 h) were designed and evaluated. By comparing the areas of characteristic peaks of different factors in each chromatogram, the optimal conditions for the extraction of the secondary metabolites from Ophiopogonis Radix were selected as 1.0 g of powder of each dried sample with 20 mL of methanol. The samples were exposed to an ultrasonication method for 30 min. Under the optimum extraction conditions, the recovery rates at the three spiking levels of the secondary metabolites, 250, 625 and 1250 ng/L, were within the acceptable range (82.6%–97.9%, Figure 2C).

### 2.3. Method Validation

The optimized HPLC-MS/MS method for quantitative analysis was validated by determining the linearity, limits of detection (LODs), limits of quantification (LOQs), precision, stability, and recovery of the targeted analytes. The results are listed in Table 2. The calibration curves showed good linearity with correlation coefficients (*r*^2^) higher than 0.9944 for all analytes in the concentration ranges. The LODs and LOQs for all analytes were less than 20 and 50 ng/mL, respectively, indicating that the developed method exhibited high sensitivity. The relative standard deviation (RSD) was used to determine the precision of the method. The precision based on the peak areas of the analytes was in the range of 2.5%–5.9% (intra-day) and 2.5%–8.3% (inter-day). All analytes exhibited good stability with an RSD for the peak area of less than 9.5%. The recovery rates of paclobutrazol from soil and Ophiopogonis Radix samples were between 84.6% and 95.1% (the average recovery was 90.2% in soil and 89.1% in Ophiopogonis Radix) with RSD values between 3.6% and 8.2%. The recovery rates of the secondary metabolites from Ophiopogonis Radix were in the range of 82.6%–97.9% (the average recovery rate was 97.1%) with RSD values between 1.8% and 12.1%, indicating that the method showed good reliability and accuracy.

The matrix effect (ME, %) was evaluated by the matrix-matched standard peak area and solvent standard peak area at a particular concentration (100 ng/mL for paclobutrazol and 625 ng/mL for the secondary metabolites). The ME was calculated using the formula: (A_1_ − A_2_) × 100/A_3_, where A_1_ is the peak area of the analyte in the spiked sample matrix, A_2_ is the peak area of the analyte in the un-spiked sample matrix, and A_3_ is the peak area of the standard in the pure solvent. The results are shown in Table 2. Signal suppression was observed for ophiopogonin D (SD), ophiopogonin D’ (SD’), methylophiopogonanone B (FNB), and ophiopogonanone C (FC). However, these slightly suppressed signals would not have interfered with the accurate determination of the targeted analytes. These results indicated that the developed method is acceptable for the quantitative analysis of the targeted analytes in samples.

### 2.4. Sample Analysis

#### 2.4.1. Residue Analysis of Paclobutrazol in Real Samples and EPI Spectra Confirmation

**Figure 3:**
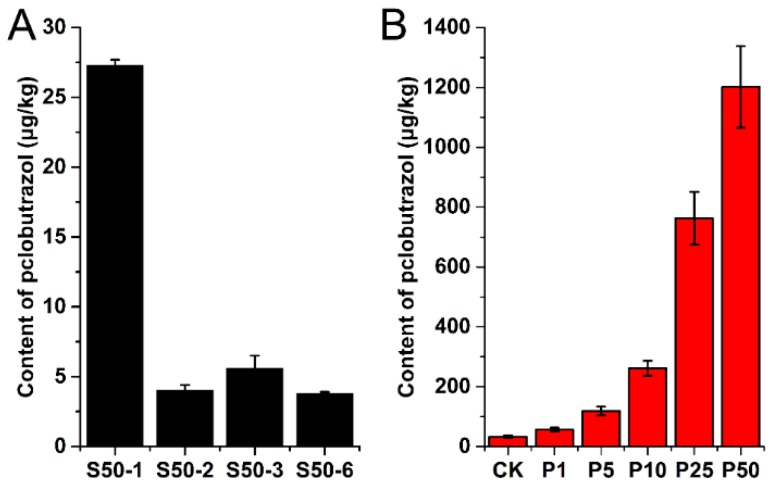
The residue levels of paclobutrazol in soil (**A**) and Ophiopogonis Radix (**B**). S50-1, -2, -3, and -6 represent soil samples from different times after the application of paclobutrazol (50g/L); P1, P5, P10, P25, and P50 represent the concentrations (g/L) of paclobutrazol.

The proposed HPLC-MS/MS method was applied to analyze the residue of paclobutrazol in Ophiopogonis Radix, soil, and water samples from Santai County (Sichuan, China). These samples included 18 Ophiopogonis Radix samples and 12 soil samples after spraying paclobutrazol as well as 3 surface water and 3 groundwater samples from the production areas of *O. japonicas*. The residue of paclobutrazol was identified by comparison of its retention time and precursor and product ions together with their ion ratios obtained from HPLC-MS/MS analysis with the data of standards. The results were confirmed using the fragment ions produced in the MRM-IDA-EPI mode. The quantitative analyses were performed by means of the internal standard (IS) method, and the results are summarized in Table 3 and Figure 3.

**Figure 4:**
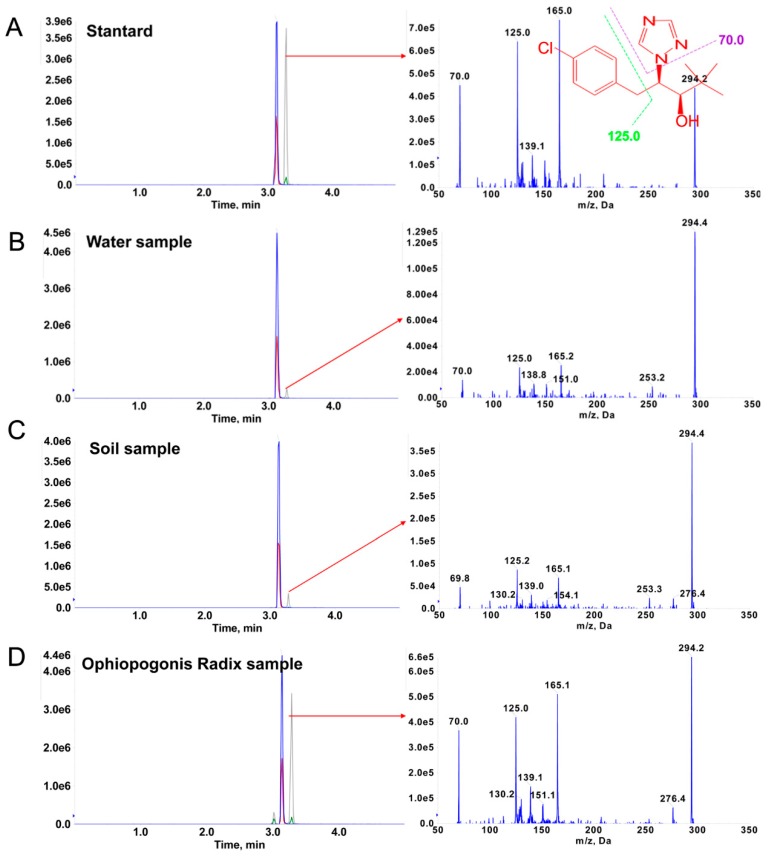
Typical selective ion chromatograms and enhanced product ion (EPI) spectra for confirmation of paclobutrazol from the standard (**A**), water (**B**), soil (**C**), and Ophiopogonis Radix (**D**) samples.

To avoid false positive results, the MRM chromatograms of the targeted compounds were triggered by IDA to get their EPI spectra, which are shown in Figure 4. The EPI characteristic fragment ions in a positive sample were compared to the MS/MS product ion spectra of the standard. The same fragments were observed in a contaminated sample, which revealed the usefulness of the current method with synchronous EPI spectra. These results validated the reliability of the established method for the determination of paclobutrazol.

The results in Table 3 revealed that paclobutrazol was detected in all samples except for two groundwater samples. The residue level of paclobutrazol varied greatly among the Ophiopogonis Radix, soil, and water samples. Paclobutrazol was detected in all Ophiopogonis Radix samples at concentrations ranging from 28.39 to 1349.51 μg/kg. Moreover, the residual level was positively correlated with the applied concentration of paclobutrazol (Figure 3B). The contents of paclobutrazol in Ophiopogonis Radix samples that applied 25 g/L and 50 g/L paclobutrazol significantly exceeded the MRL in crops, vegetables, and fruits (0.05–0.5 mg/kg) according to GB 2763-2016 [26]. It is noteworthy that the residue of paclobutrazol was also detected in the control group of Ophiopogonis Radix. This may be due to the soil in the planting area being contaminated by the previous use of paclobutrazol or caused by paclobutrazol from the treated group transferring by the flow of rainwater. Earlier research indicated that paclobutrazol can be enriched in soil and is comparatively resistant to degradation [27,28]. In this study, soil samples from the paclobutrazol (50 g/L)-treated group were collected and detected at 1, 2, 3 and 6 months after spraying. The results showed that with an increase in time, the concentration of paclobutrazol in soil gradually decreased (Figure 3A). Although the concentration of paclobutrazol in soil decreased significantly within 2 months after spraying, the concentration changed slightly between 2 and 6 months. From our results, we know that paclobutrazol remains in soil for a long time after spraying, but it remains to be confirmed whether the change in the residue levels is due to degradation or dilution in the soil. Samples of surface water and groundwater were collected from Santai County (Sichuan, China), which is the main production area of *O. japonicus*. It should be noted that the surface water represents pond water and the groundwater represents well water (to a depth of 20 m) in this study. The analysis of three surface water samples showed the presence of paclobutrazol with a residual concentration ranging from 1.08 μg/L to 1.62 μg/L. The concentration of paclobutrazol in groundwater water was lower than the LOD and was only detected in one sample.

GB2763-2016 stipulates that the MRL of paclobutrazol in rice, wheat, apple, rapeseed oil, and peanut kernel is 0.5 mg/kg, that of rapeseed is 0.2 mg/kg, and that of soybean and mango is 0.05 mg/kg. The United States, the European Union, and South Korea stipulate that the residues of paclobutrazol in vegetables should be below the detection limit. Paclobutrazol is banned in Sweden. The risk assessment and the residual decline pattern of paclobutrazol were investigated, showing that triazoles (paclobutrazol) and structurally similar chemicals are potential endocrine disruptors. However, paclobutrazol is overused for the treatment of *O. japonicus* and other medicinal plants. At present, PGR residues in TCM are not regularly monitored in China and other countries. From the above experimental results, it can be seen that paclobutrazol has a long residual period in soil, and water resources are also polluted. Ultimately, paclobutrazol may have a toxic effect on the ecological environment of the production area and even on the health of local residents. In addition, as an important TCM, the excessive paclobutrazol residues in Ophiopogonis Radix may threaten the health of TCM users.

#### 2.4.2. Quantitative Analysis of the Secondary Metabolites in Ophiopogonis Radix Samples

The established analytical method was applied to detect nine secondary metabolites, including four steroidal saponins and five flavonoids in eighteen Ophiopogonis Radix samples after spraying with paclobutrazol. The targeted analytes were identified on the basis of comparison of their retention times, precursors, and product ions obtained from LC-MS/MS analysis of the standard compounds. The quantitative analysis was performed by means of the external standard method, and the data are summarized in Table 4.

**Figure 5:**
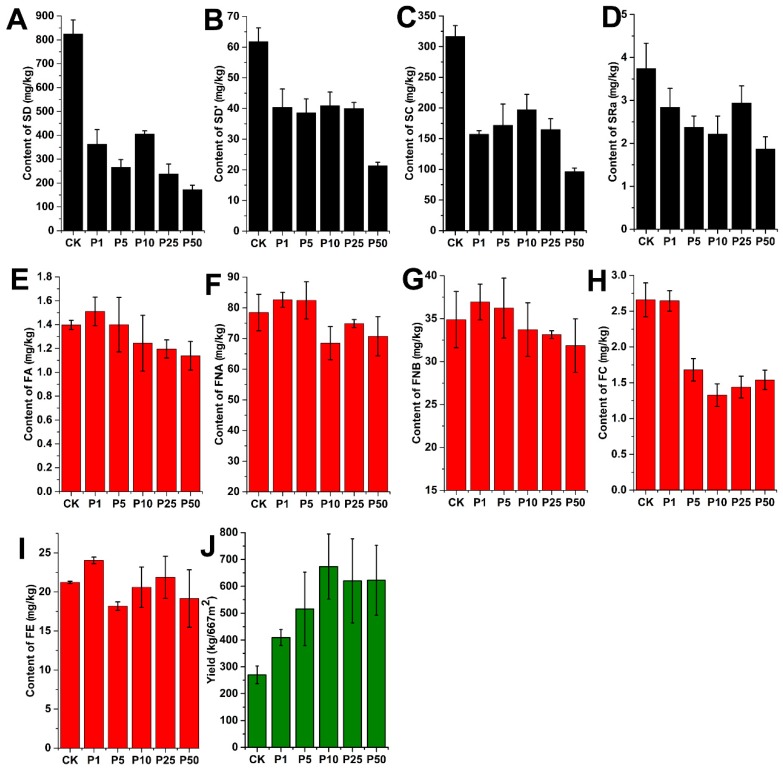
The contents of nine secondary metabolites (**A**–**I**) in Ophiopogonis Radix and the yield of Ophiopogonis Radix in different treated groups (**J**): SD (**A**), SD’ (**B**), SC (**C**), SRa (**D**), FA (**E**), FNA (**F**), FNB (**G**), FC (**H**), and FE (**I**).

The results showed that, in all Ophiopogonis Radix samples, the content of SD was the highest in all targeted analytes. Among the detected four steroidal saponins, the content, from high to low, occurred in SD, SC, SD’, and ophiopogon Ra (SRa). Of the five detected flavonoids, methylophiopogonanone A (FNA) was found to have the highest content in Ophiopogonis Radix, followed by FNB, ophiopogonanone E (FE), FC, and methylophiopogonone A (FA). According to the comparative analysis of the control group and the different treated groups, it was found that paclobutrazol significantly changed the content of secondary metabolites in Ophiopogonis Radix (Figure 5). In particular, the effect on SD was significant; the mean content of SD in the control group was 824.87 mg/kg, while there was only 172.50 mg/kg in the treated group (50 g/L paclobutrazol). As shown in Figure 5A, the decrease in SD was related to the concentration of paclobutrazol. Moreover, the contents of SD in all treated groups were much lower than that in the control group. The content was reduced by more than one-fold after the application of paclobutrazol. These results indicated that paclobutrazol has an obvious inhibitory action on the accumulation of SD in *O. japonicus*. In addition, the effects of paclobutrazol on the other three steroidal saponins were also significant. The mean contents of SC, SD’, and SRa in the control group were 316.88, 61.81, and 3.71 mg/kg, while they were only 96.15, 21.32, and 1.87 mg/kg in the treated group (50 g/L paclobutrazol).

Compared with that of steroidal saponins, the effect of paclobutrazol on flavonoids was relatively weak. As shown in Figure 5H, although FC was affected by paclobutrazol and the content was significantly decreased from 2.66 to 1.33 mg/kg, we did not find any significant changes in the contents of FA, FNA, FNB, or FE after paclobutrazol treatment. However, when treated with a low concentration of paclobutrazol (1 g/L), the contents of FA, FNA, FNB, and FE were slightly higher than those of the control group. These results showed that paclobutrazol is not conducive to the accumulation of the secondary metabolites in Ophiopogonis Radix, and it ultimately affects the quality of Ophiopogonis Radix. Conversely, a number of studies in a vast number of plants have been performed and confirmed that PGR is a proficient elicitor that stimulates the production of secondary plant metabolites, such as triterpenoids, flavonoids, alkaloids, etc. [29]. Therefore, a plant’s reaction to PGRs may differ depending on the PGR classification or the plant itself. Ophiopogonis Radix is an important traditional Chinese herbal medicine and functional food, and the negative regulation on the secondary metabolites of Ophiopogonis Radix by paclobutrazol may affect its effectiveness as a drug.

However, in order to increase the yield of Ophiopogonis Radix and shorten the planting period, paclobutrazol is widely used in production. In this study, we also determined the yield of Ophiopogonis Radix after treatment with paclobutrazol. Specifically, samples of Ophiopogonis Radix were collected from three duplicate experimental districts of each treated group. Then, the weight of fresh Ophiopogonis Radix was determined after processing. Finally, the yield of fresh Ophiopogonis Radix from each experimental district was converted into the yield per 667 m^2^. The results showed that the use of paclobutrazol could greatly increase the yield (up to 1–2 times) of Ophiopogonis Radix (Figure 5J).

## 3. Materials and Methods

### 3.1. Chemicals and Reagents

The pesticide standards of paclobutrazol and forchlorfenuron were purchased from Sigma-Aldrich Corporation (St. Louis, Mo, USA). Forchlorfenuron was used as an IS in the residue analysis. Standard compounds of FNA, FA, FNB, FC, SD, SD’, SRa, and SC were provided by Chengdu Must Bio-technology Co. Ltd. (Chengdu, China). FE was obtained from Shanghai Yiling Co. Ltd. (Shanghai, China). The purities of these reference compounds were all over 98.0%, except for FC, which was 92%, by area percent using HPLC-ELSD or HPLC-UV analysis. The chemical structures and related information are summarized in Table S1 (Supplementary Materials). HPLC-grade acetonitrile and methanol were purchased from Fisher Scientific (Fair Lawn, NJ, USA). Other reagents and chemicals were of analytical grade and were bought from Sinopharm Chemical Regent Beijing Co., Ltd. (Beijing, China). Ultrapure water was prepared in our laboratory using a Milli-Q water purification system (Millipore, Milford, MA, USA).

### 3.2. Plant Materials and Paclobutrazol Treatment

*O. japonicus* was planted on flat farmland (667 m^2^) in Santai County (Sichuan, China) from April 2018 to April 2019. The plant density was about 100 plants per m^2^. In October 2018, the land was randomly divided into several experimental districts with an area of about 30 m^2^. Paclobutrazol SC (25%) from Jiangsu Jianpai Agrochemical Co., Ltd. (Jiangsu, China) was diluted with water into 50, 25, 10, 5, and 1 g/L solutions separately. Then, the different concentrations were uniformly sprayed on the leaves of plants with small sprayers. Each concentration was used in three randomized experimental districts. The control group did not receive any treatment. After spraying paclobutrazol for 1, 2, 3, and 6 months (harvest time), 12 soil samples, about 500 g per sample, were collected (to a depth of 20 cm) from the 50 g/L treated group. At harvest time, tuberous roots of *O. japonicas* from the control group and treated groups (18 samples in total) were collected for yield and quality evaluation. In addition, 3 batches of surface water and 3 batches of groundwater, about 500 mL per sample, were collected from the production areas of *O. japonicas* at harvest time. The samples of Ophiopogonis Radix and soil were pulverized and homogenized after drying and then sealed in Ziplock bags. All samples were stored at 4 °C prior to analysis. The *O. japonicus* (L.f.) Ker-Gawl plants were identified by Prof. Xiaojun Ma, and the voucher specimens of the plant and the samples were deposited in our laboratory (Institute of Medicinal Plant Development, Chinese Academy of Medical Sciences and Peking Union Medical College, Beijing, China).

### 3.3. Preparation of Standard Solutions

The stock solution of paclobutrazol was prepared at a concentration of 1 mg/mL in acetonitrile. Then, standard working solutions of different concentrations were obtained by dissolving the stock solutions with acetonitrile to construct calibration curves. All working solutions contained 100 ng/mL IS. All solutions were filtered through a 0.22 μm membrane and stored at 4 °C prior to analysis.

Stock solutions of FNA, FA, FNB, FC, FE, SD, SD’, SRa, and SC were individually prepared at a concentration of 1 mg/mL in methanol. The stock solutions of the standards were further diluted with a mixed solvent of acetonitrile–water (50:50, *v*/*v*) to produce combined standard working solutions. All solutions were stored in a refrigerator at 4 °C for analysis. Six concentrations of the solution were analyzed, and then the calibration curves were constructed by plotting the peak area versus the concentration of the analyte.

### 3.4. Preparation of Sample Solutions

For the residue analysis of paclobutrazol, the homogenized Ophiopogonis Radix (1.0 g) and soil (5.0 g) samples were accurately weighed and separately placed into 25 mL centrifuge tubes and mixed with 10 mL of acetonitrile containing 1% formic acid. The mixtures were vortexed for 30 s and then subjected to ultrasonic extraction for 10 min. Then, 1.0 mL of the upper layer extract was transferred to a 2 mL Teflon centrifuge tube containing 150 mg anh. MgSO_4_ and 50 mg PSA. The mixture was vortexed for 30 s and centrifuged at 10,000× *g* for 5 min. Finally, 0.5 mL IS (200 ng/mL) dissolved with the acetonitrile–water mixed solution (50:50, *v*/*v*) was added to 0.5 mL of the supernatant and vortexed for 30 s. Then, the mixture was filtered through a 0.22 μm membrane prior to HPLC-MS/MS analysis. The water (20 mL) samples were accurately measured and condensed under reduced pressure using a rotary evaporator. The residue was dissolved in 2 mL of acetonitrile–water mixture (50:50, *v*/*v*). Then, 1.0 mL of the upper layer extract was transferred to a 2 mL Teflon centrifuge tube containing 150 mg anh. MgSO_4_ and 50 mg PSA. The subsequent processing is consistent with the above method.

The homogenized Ophiopogonis Radix (1.0 g) samples were accurately weighed and ultrasonically extracted with 20 mL of methanol for 30 min for the analysis of the secondary metabolites (including flavonoids and steroidal saponins). Then, 1.0 mL of the upper layer extract was one-fold diluted with acetonitrile. The mixture was vortexed for 30 s and filtered through a 0.22 μm membrane prior to HPLC-MS/MS analysis.

### 3.5. Apparatus and Analytical Conditions

All experiments were carried out using an Agilent 1260 series HPLC system (Agilent Technologies, Palo Alto, CA, USA) coupled to a QTRAP^®^ 4500 mass spectrometer (AB SCIEX, Foster City, CA, USA) via an ESI interface. Applied Biosystems Analyst software (version 1.6) was used to control the HPLC QTrap-MS/MS system and for data acquisition and processing.

The chromatographic separation of paclobutrazol was performed on an Agilent Poroshell 120 EC C_18_ column (100 mm × 3.0 mm, 2.7 μm; Agilent, Palo Alto, CA, USA) by gradient elution at a flow rate of 0.3 mL/min. The mobile phase consisted of water containing 0.1% formic acid (A) and acetonitrile (B) with the following gradient procedure: 0–1.00 min, 5% B; 1.01–2.50 min, 85%–90% B; 2.50–3.00 min, 90%–5% B; then hold at 5% B for 2 min. The injection volume was 10 μL. MS detection was conducted with the ESI source in positive mode. Curtain gas (CUR), nebulizer gas (GS1), and auxiliary gas (GS2) were set at 35, 50, and 50 psi, respectively. The ion spray voltage (IS) was 5500 V, and the source temperature was 500 °C. MRM mode was adopted for quantitation. The dwell time was 20 ms for each MRM transition. An IDA experiment was conducted to automatically trigger an EPI scan and synchronously acquire supplementary confirmation of the targeted analytes [30].

The secondary metabolites of Ophiopogonis Radix were separated on an Agilent Poroshell 120 SB C18 column (100 mm × 2.1 mm, 2.7 μm; Agilent, Palo Alto, CA, USA) by using a binary mobile phase composed of water containing 0.1% formic acid (A) and acetonitrile (B) with the following gradient elution program: 0–4.0 min, 45%–70% B; 4.1–5.0 min, 100% B; 5.1–8.0 min, 45% B. The injection volume was 2 μL. All targeted analytes were determined by MRM in positive and negative ion modes. CUR, GS1, and GS2 were set at 35, 50, and 50 psi, respectively. The ion spray voltage (IS) was 5500 V, and the source temperature was 500 °C. MRM mode was adopted for quantitation. The dwell time was 50 ms for each MRM transition.

### 3.6. Method Validation

According to the EU guidance document on residue analytical methods [31] and the International Conference on Harmonization (ICH) guidelines on analytical method validation [32], the method was validated in terms of its linearity, selectivity, sensitivity, precision (intra- and inter-day variability), stability, accuracy, and ME. Calibration curves were constructed using the peak area (Y) versus the analyte concentration (X). Each calibration curve was constructed with at least six appropriate concentration levels in triplicate. The LOD and LOQ for each analyte were determined at S/N ratios of about 3 and 10, respectively. Precision was determined by replicated analyses (*n* = 6) of standard samples within a day (intra-day variation) and on three consecutive days (inter-day variation). The accuracy of the method was assessed by adding the targeted analytes at three different concentrations to the sample that had previously been analyzed. The stability of the sample solution was tested at room temperature. The sample solution was analyzed in triplicate every 12 h within 2 days. The ME was assessed by adding the targeted analytes at their respective concentrations to the matrix solutions of sample.

## 4. Conclusions

Paclobutrazol was detected in Ophiopogonis Radix, and the residual level was positively correlated with the applied concentration. The residual level of paclobutrazol in soil gradually decreased over time, but the residual level was still significant after 6 months of spraying. In addition, paclobutrazol was found in a groundwater sample and in all surface water samples. All of these results indicated that paclobutrazol not only affects the safety of Ophiopogonis Radix but also causes serious environmental pollution problems. In addition, paclobutrazol significantly decreased the concentrations of secondary metabolites, including steroidal saponins and flavonoids, in Ophiopogonis Radix, which may affect the effectiveness of Ophiopogonis Radix as a TCM. What is really advantageous is that paclobutrazol can not only shorten the planting cycle of *O. japonicus* and increase its yield, but it can also bring intuitive economic benefits to farmers. Therefore, when determining whether to apply paclobutrazol or not, the advantages and disadvantages should be fully evaluated before making a decision.

## Figures and Tables

**Table 1:** The optimized MS/MS parameters for the targeted analytes.

No.	Analytes	Precursor Ion (*m*/*z*)		Product Ions (*m*/*z*)				Ionization Mode	Retention Time (min)
		Q1	DP (V)	PI *^q^*	CE (V)	PI *^i^*	CE (V)		
1	Forchlorfenuron	248	22	129	30	92.9	35	ESI^+^	3.13
2	Paclobutrazol	294.1	60	69.9	30	124.8	25	ESI^+^	3.28
3	Ophiopogon Ra (SRa)	719.6	220	393.2	45	411.4	40	ESI^+^	1.8
4	Ophiopojaponin C (SC)	869.5	−220	737.4	−50	205.6	−50	ESI^−^	2.48
5	Ophiopogonanone E (FE)	359.2	−50	153.9	−35	208	−35	ESI^−^	3.98
6	Ophiopogonin D (SD)	853.6	−220	575.3	−50	721.6	−55	ESI^−^	4.14
7	Ophiopogonin D’ (SD’)	853.6	−220	204.9	−55	721.6	−55	ESI^−^	4.41
8	Methylophiopogonone A (FA)	339.2	−68	131	−55	217.2	−55	ESI^−^	5.51
9	Methylophiopogonanone A (FNA)	341.3	−68	178	−45	206	−38	ESI^−^	5.7
10	Methylophiopogonanone B (FNB)	327	−60	206	−35	178	−40	ESI^−^	5.89
11	Ophiopogonanone C (FC)	355.2	−60	193.1	−40	164.2	−45	ESI^−^	6.61

DP: declustering potential; CE: collision energy; ESI: electrospray ionization; Q1: precursor ion; PI: product ions; *^q^*: for quantification; *^i^*: for identification.

**Table 2:** Method validation results including the linearity, limit of detection (LOD) and limit of quantification (LOQ), precision (relative standard deviation, RSD, %), recovery (%), stability (RSD, %) and matrix effect (ME, %).

Analytes	Linearity		LOQ (ng/mL)	LOD (ng/mL)	Precision (RSD, %)		Recovery% (RSD, %)			Stability (RSD, %)	ME (%)
	*r* ^2^	Range (ng/mL)			Intra-Day	Inter-Day	Low Level	Medium Level	High Level		
Paclobutrazol	0.9993	2–100	0.5	0.2	5.0	4.3	Soil sample			5.7	94.9
							90.2 (8.0)	85.2 (8.2)	95.1 (3.6)		
							Ophiopogonis Radix				91.0
							92.1 (4.6)	84.6 (4.3)	90.6 (7.4)		
SRa	0.9963	50–1000	50.0	20.0	5.6	6.2	84.4 (10.5)	95.5 (4.6)	92.6 (6.9)	9.5	95.2
SC	0.9998	10–1000	10.0	4.0	4.4	6.3	93.6 (1.8)	83.0 (9.1)	92.6 (6.9)	4.5	99.1
FE	0.9995	5–500	2.0	0.8	2.5	3.5	93.7 (4.4)	92.5 (10.4)	96.7 (2.7)	2.8	90.8
SD	0.9944	50–1000	30.0	10.0	5.9	7.3	86.3 (2.3)	84.4 (5.4)	88.6 (6.1)	8.1	86.4
SD’	0.9998	50–1000	30.0	10.0	4.2	4.8	85.0 (9.4)	90.1 (8.9)	97.6 (3.0)	2.5	84.3
FA	0.9998	5–500	5.0	2.0	3.1	5.1	84.3 (11.1)	93.3 (9.1)	97.9 (1.8)	5.7	96.7
FNA	0.9996	5–500	5.0	2.0	4.0	2.5	95.7 (2.3)	95.3 (2.3)	97.4 (2.1)	3.8	93.9
FNB	0.9995	5–500	2.0	0.8	5.1	8.3	92.2 (2.5)	89.2 (5.5)	95.8 (2.7)	6.0	84.8
FC	0.9998	5–500	5.0	2.0	4.6	3.5	82.6 (10.8)	88.1 (12.1)	92.0 (8.8)	5.4	85.9

**Table 3:** Paclobutrazol residue levels in Ophiopogonis Radix, soil, and water samples.

Ophiopogonis Radix Sample	Residue Level (μg/kg)	Soil Sample	Residue Level (μg/kg)	Water Sample	Residue Level (μg/L)
CK1	31.67	S50-1-1	26.96	Groundwater 1	ND
CK2	28.39	S50-1-2	27.17	Groundwater 2	ND
CK3	37.66	S50-1-3	27.72	Groundwater 3	<LOQ
P1-1	58.94	S50-2-1	4.39	Surface water 1	1.16
P1-2	49.72	S50-2-2	3.65	Surface water 2	1.62
P1-3	61.38	S50-2-3	4.06	Surface water 3	1.08
P5-1	125.03	S50-3-1	6.46		
P5-2	101.94	S50-3-2	5.68		
P5-3	128.76	S50-3-3	4.69		
P10-1	269.21	S50-6-1	3.92		
P10-2	233.74	S50-6-2	3.69		
P10-3	282.00	S50-6-3	3.74		
P25-1	675.57				
P25-2	851.59				
P25-3	760.64				
P50-1	1081.37				
P50-2	1174.25				
P50-3	1349.59				

P1, P5, P10, P25, and P50 represent the concentrations (g/L) of paclobutrazol; S50-1, -2, -3, and -6 represent soil samples from different times after the application of paclobutrazol (50g/L); ND: not detected.

**Table 4:** The contents (mg/kg) of nine secondary metabolites in Ophiopogonis Radix samples.

Samples	SRa	FNB	FA	FNA	FC	FE	SD	SD’	SC
CK1	3.37	33.08	1.35	78.64	2.41	21.30	781.35	56.72	297.40
CK2	3.43	38.65	1.41	84.30	2.88	21.30	891.19	65.02	331.17
CK3	4.42	32.88	1.43	72.42	2.68	21.06	802.07	63.68	322.08
P1-1	2.78	39.23	1.59	79.77	2.70	23.90	341.97	38.39	155.84
P1-2	2.43	35.19	1.57	84.30	2.75	24.51	315.03	35.59	151.95
P1-3	3.31	36.35	1.37	83.73	2.48	23.69	431.09	47.09	163.64
P5-1	2.32	40.19	1.65	89.39	1.85	18.80	292.23	33.31	149.35
P5-2	2.15	33.65	1.22	78.64	1.63	17.98	230.05	41.47	154.55
P5-3	2.66	34.81	1.33	79.21	1.55	17.74	275.65	40.94	211.69
P10-1	2.33	30.96	1.49	67.89	1.40	23.56	397.93	46.02	180.52
P10-2	1.75	37.12	1.22	74.12	1.44	19.49	395.85	38.13	225.97
P10-3	2.57	33.08	1.02	63.37	1.15	18.74	420.73	38.53	185.71
P25-1	2.92	32.88	1.26	74.12	1.26	22.60	191.71	40.40	166.23
P25-2	2.55	32.88	1.11	74.12	1.54	18.87	250.78	37.86	146.75
P25-3	3.35	33.65	1.22	76.38	1.52	24.14	271.50	41.74	181.82
P50-1	1.55	35.19	1.22	78.08	1.66	23.38	167.67	20.74	95.19
P50-2	1.95	31.35	1.00	67.89	1.39	16.79	157.10	20.60	90.65
P50-3	2.11	29.04	1.20	66.20	1.57	17.26	192.75	22.61	102.60

P1, P5, P10, P25, and P50 represent the concentrations (g/L) of paclobutrazol.

## Supplementary Materials

The following are available online. Table S1. The chemical structures and related information of the targeted analytes.

## Abbreviations

FNA	methylophiopogonanone A
FA	methylophiopogonone A
FNB	methylophiopogonanone B
FC	ophiopogonanone C
FE	ophiopogonanone E
SD	ophiopogonin D
SD’	ophiopogonin D’
SRa	ophiopogon Ra
SC	ophiopojaponin C
MRM	multiple reaction monitoring
TCM	traditional Chinese medicine
PGR	plant growth regulator
MRM	multiple reaction monitoring
IDA	Information-dependent acquisition
EPI	enhanced product ion
ME	matrix effect
MRL	maximum residue limit
