# Supplementary material for: Residue of Paclobutrazol and Its Regulatory Effects on the Secondary Metabolites of Ophiopogon japonicas
Source: Molecules. 2019 Sep 27;24(19):3504. doi: 10.3390/molecules24193504 (PMC6804066; doi:10.3390/molecules24193504)
Supplement: Supplementary file 1 [file molecules-24-03504-s001.pdf]

Table S1. The chemical structures and related information of the targeted analytes.

Analytes	Chemical Structures	CAS Number	Molecular Formula	Molecular Weight	Other Information
Paclobutrazol	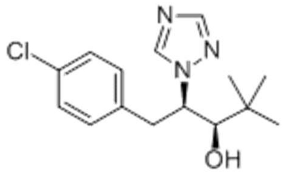	76738-62-0	C15H20ClN3O	293.79	$[M + H]^+$: 294.1<br>Log Kow: 3.2<br>IUPAC Name: (2*R*,3*R*)-1-(4-chlorophenyl)-4,4-dimethyl-2-(1,2,4-triazol-1-yl)pentan-3-ol<br>InChI: 1S/C15H20ClN3O/c1-15(2,3)14(20)13(19-10-17-9-18-19)8-11-4-6-12(16)7-5-11/h4-7,9-10,13-14,20H,8H2,1-3H3/t13-,14+/m1/s1<br>SMILES: CC(C)(C)C(C(CC1=CC=C(C=C1)Cl)N2C=NC=N2)O
Forchlorfenuron	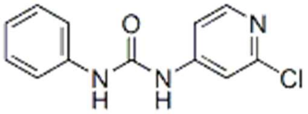	68157-60-8	C12H10ClN3O	247.68	$[M + H]^+$: 248.3<br>Log Kow: 3.2<br>IUPAC Name: 1-(2-chloropyridin-4-yl)-3-phenylurea<br>InChI: 1S/C12H10ClN3O/c13-11-8-10(6-7-14-11)16-12(17)15-9-4-2-1-3-5-9/h1-8H,(H2,14,15,16,17)<br>SMILES: C1=CC=C(C=C1)NC(=O)NC2=CC(=NC=C2)Cl
Methylophiopogonanone A	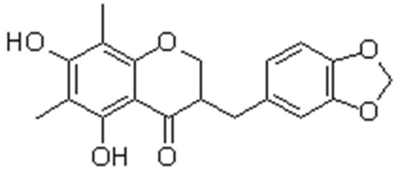	74805-92-8	C19H18O6	342.34	$[M - H]^-$: 341.3<br>IUPAC Name: 3-(1,3-benzodioxol-5-ylmethyl)-5,7-dihydroxy-6,8-dimethyl-2,3-dihydrochromen-4-one<br>InChI: 1S/C19H18O6/c1-9-16(20)10(2)19-15(17(9)21)18(22)12(7-23-19)5-11-3-4-13-14(6-11)25-8-24-13/h3-4,6,12,20-21H,5,7-8H2,1-2H3

					SMILES:<br>CC1=C(C(=C2C(=C1O)C(=O)C(CO2)CC3=CC4=C(C=C3)OCO4)C)O
Methylophiopogonone A	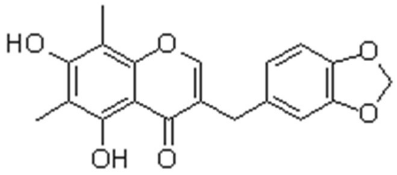	74805-90-6	C19H16O6	340.32	[M − H]$^-$: 339.2<br>IUPAC Name: 3-(1,3-benzodioxol-5-ylmethyl)-5,7-dihydroxy-6,8-dimethylchromen-4-one<br>InChI: 1S/C19H16O6/c1-9-16(20)10(2)19-15(17(9)21)18(22)12(7-23-19)5-11-3-4-13-14(6-11)25-8-24-13/h3-4,6-7,20-21H,5,8H2,1-2H3<br>SMILES:<br>CC1=C(C(=C2C(=C1O)C(=O)C(=CO2)CC3=CC4=C(C=C3)OCO4)C)O
Methylophiopogonanone B	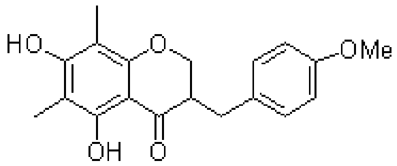	74805-91-7	C19H20O5	328.35	[M − H]$^-$: 327.0<br>IUPAC Name: (3*R*)-5,7-dihydroxy-3-[(4-methoxyphenyl)methyl]-6,8-dimethyl-2,3-dihydrochromen-4-one<br>InChI: 1S/C19H20O5/c1-10-16(20)11(2)19-15(17(10)21)18(22)13(9-24-19)8-12-4-6-14(23-3)7-5-12/h4-7,13,20-21H,8-9H2,1-3H3/t13-/m1/s1<br>SMILES:<br>CC1=C(C(=C2C(=C1O)C(=O)C(CO2)CC3=CC=C(C=C3)OC)C)O
Ophiopogonanone C	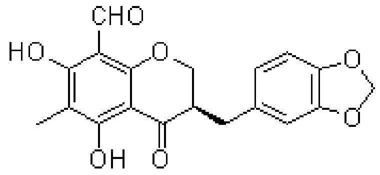	477336-75-7	C19H16O7	356.32	[M − H]$^-$: 355.2<br>IUPAC Name: 3-(1,3-benzodioxol-5-ylmethyl)-5,7-dihydroxy-6-methyl-4-oxo-2,3-dihydrochromene-8-carbaldehyde<br>InChI: 1S/C19H16O7/c1-9-16(21)12(6-20)19-15(17(9)22)18(23)11(7-24-19)4-10-2-3-13-14(5-10)26-8-25-13/h2-3,5-6,11,21-22H,4,7-8H2,1H3<br>SMILES:<br>CC1=C(C(=C2C(=C1O)C(=O)C(CO2)CC3=CC4=C(C=C3)OCO4)C=O)O
Ophiopogonanone E		588706-66-5	C19H20O7	360.35	[M − H]$^-$: 359.2

	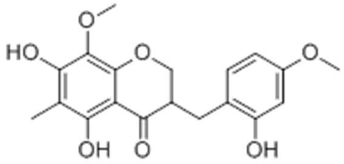				IUPAC Name: 5,7-dihydroxy-3-[(2-hydroxy-4-methoxyphenyl)methyl]-8-methoxy-6-methyl-2,3-dihydrochromen-4-one<br>InChI: 1S/C19H20O7/c1-9-15(21)14-17(23)11(8-26-18(14)19(25-3)16(9)22)6-10-4-5-12(24-2)7-13(10)20/h4-5,7,11,20-22H,6,8H2,1-3H3<br>SMILES: CC1=C(C2=C(C(=C1O)OC)OCC(C2=O)CC3=C(C=C(C=C3)OC)O)O
Ophiopogonin D	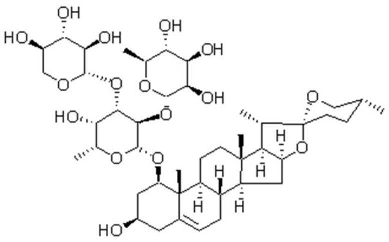	41753-55-3	C44H70O16	855.021	$[M - H]^-$: 853.6<br>IUPAC Name: (2*S*,3*R*,4*R*,5*R*,6*S*)-2-[(2*R*,3*R*,4*S*,5*S*,6*R*)-5-hydroxy-2-[(1*S*,2*S*,4*S*,5′*R*,6*R*,7*S*,8*R*,9*S*,12*S*,13*R*,14*R*,16*R*)-16-hydroxy-5′,7,9,13-tetramethylspiro[5-oxapentacyclo[10.8.0.0^{2,9}.0^{4,8}.0^{13,18}]icos-18-ene-6,2′-oxane]-14-yl]oxy-6-methyl-4-[(2S,3R,4S,5R)-3,4,5-trihydroxyoxan-2-yl]oxyoxan-3-yl]oxy-6-methyloxane-3,4,5-triol<br>InChI: 1S/C44H70O16/c1-18-9-12-44(54-16-18)19(2)30-28(60-44)15-26-24-8-7-22-13-23(45)14-29(43(22,6)25(24)10-11-42(26,30)5)57-41-38(59-40-36(52)34(50)31(47)20(3)55-40)37(32(48)21(4)56-41)58-39-35(51)33(49)27(46)17-53-39/h7,18-21,23-41,45-52H,8-17H2,1-6H3/t18-,19+,20+,21-,23-,24-,25+,26+,27-,28+,29-,30+,31+,32+,33+,34-,35-,36-,37+,38-,39+,40+,41+,42+,43+,44-/m1/s1<br>SMILES: CC1CCC2(C(C3C(O2)CC4C3(CCC5C4CC=C6C5(C(CC(C6)O)OC7C(C(C(C(O7)C)O)OC8C(C(C(CO8)O)O)O)OC9C(C(C(C(O9)C)O)O)O)C)C)C)OC1

Ophiopogonin D′	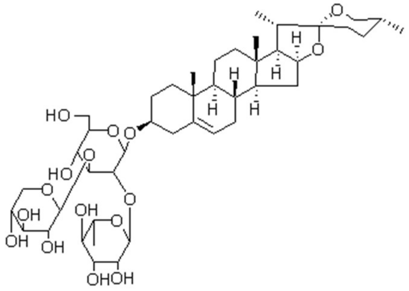	65604-80-0	$C_{44}H_{70}O_{16}$	855.01	$[M-H]^-$: 853.6<br>IUPAC Name: (2*S*,3*R*,4*R*,5*R*,6*S*)-2-<br>[(2*R*,3*R*,4*S*,5*R*,6*R*)-5-hydroxy-6-<br>(hydroxymethyl)-2-<br>[(1*S*,2*S*,4*S*,5′*R*,6*R*,7*S*,8*R*,9*S*,12*S*,13*R*,16*S*)-<br>5′,7,9,13-tetramethylspiro[5-<br>oxapentacyclo[10.8.0.$0^{2,9}$.$0^{4,8}$.$0^{13,18}$]icos-18-ene-<br>6,2′-oxane]-16-yl]oxy-4-[(2S,3R,4S,5R)-3,4,5-<br>trihydroxyoxan-2-yl]oxyoxan-3-yl]oxy-6-<br>methyloxane-3,4,5-triol<br>InChI: 1S/C44H70O16/c1-19-8-13-44(54-17-<br>19)20(2)30-28(60-44)15-26-24-7-6-22-14-23(9-<br>11-42(22,4)25(24)10-12-43(26,30)5)56-41-<br>38(59-40-36(52)34(50)31(47)21(3)55-<br>40)37(33(49)29(16-45)57-41)58-39-<br>35(51)32(48)27(46)18-53-39/h6,19-21,23-41,45-<br>52H,7-18H2,1-<br>5H3/t19-,20+,21+,23+,24-,25+,26+,27-,28+,29-,3<br>0+,31+,32+,33-,34-,35-,36-,37+,38-,39+,40+,41-,4<br>2+,43+,44-/m1/s1<br>SMILES:<br>CC1CCC2(C(C3C(O2)CC4C3(CCC5C4CC=C<br>6C5(CCC(C6)OC7C(C(C(C(O7)CO)O)OC8C(<br>C(C(CO8)O)O)O)OC9C(C(C(C(O9)C)O)O)O)<br>C)C)C)OC1
Ophiopogon Ra	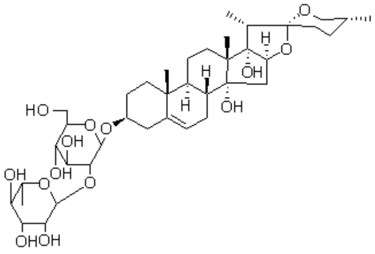	128502-94-3	$C_{39}H_{62}O_{14}$	754.9	$[M-2H_2O+H]^+$: 719.6<br>IUPAC Name: (2*S*,3*R*,4*R*,5*R*,6*S*)-2-<br>[(2*R*,3*R*,4*S*,5*S*,6*R*)-2-<br>[(1*R*,2*R*,4*S*,5′*R*,6*R*,7*S*,8*S*,9*S*,12*S*,13*R*,16*S*)-2,8-<br>dihydroxy-5′,7,9,13-tetramethylspiro[5-<br>oxapentacyclo[10.8.0.$0^{2,9}$.$0^{4,8}$.$0^{13,18}$]icos-18-ene-<br>6,2′-oxane]-16-yl]oxy-4,5-dihydroxy-6-<br>(hydroxymethyl)oxan-3-yl]oxy-6-<br>methyloxane-3,4,5-triol<br>InChI: 1S/C39H62O14/c1-18-8-13-38(48-17-<br>18)20(3)39(47)26(53-38)15-37(46)24-7-6-21-14-

					22(9-11-35(21,4)23(24)10-12-36(37,39)5)50-34-32(30(44)28(42)25(16-40)51-34)52-33-31(45)29(43)27(41)19(2)49-33/h6,18-20,22-34,40-47H,7-17H2,1-5H3/t18-,19+,20-,22+,23+,24-,25-,26+,27+,28-,29-,30+,31-,32-,33+,34-,35+,36+,37-,38-,39-/m1/s1<br>SMILES:<br>CC1CCC2(C(C3(C(O2)CC4(C3(CCC5C4CC=C6C5(CCC(C6)OC7C(C(C(C(O7)CO)O)O)OC8C(C(C(C(O8)C)O)O)O)C)C)O)O)C)OC1
Ophiopojaponin C	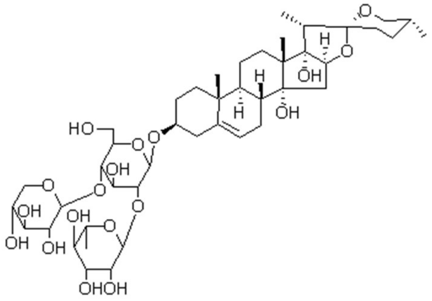	911819-08-4	$C_{44}H_{70}O_{18}$	887.02	$[M - H_2O - H]^-$: 869.5<br>IUPAC Name:<br>[(1*S*,2*S*,4*S*,5′*R*,6*R*,7*S*,8*R*,9*S*,12*S*,13*R*,14*R*,16*R*)-14-[(2*S*,3*R*,4*S*,5*S*,6*R*)-5-hydroxy-6-methyl-3-[(2*S*,3*R*,4*R*,5*R*,6*S*)-3,4,5-trihydroxy-6-methyloxan-2-yl]oxy-4-[(2*S*,3*R*,4*S*,5*R*)-3,4,5-trihydroxyoxan-2-yl]oxyoxan-2-yl]oxy-5′,7,9,13-tetramethylspiro[5-oxapentacyclo[10.8.0.$0^{2,9}$.$0^{4,8}$.$0^{13,18}$]icos-18-ene-6,2′-oxane]-16-yl] acetate<br>InChI: 1S/C46H72O17/c1-19-10-13-46(56-17-19)20(2)32-30(63-46)16-28-26-9-8-24-14-25(59-23(5)47)15-31(45(24,7)27(26)11-12-44(28,32)6)60-43-40(62-42-38(54)36(52)33(49)21(3)57-42)39(34(50)22(4)58-43)61-41-37(53)35(51)29(48)18-55-41/h8,19-22,25-43,48-54H,9-18H2,1-7H3/t19-,20+,21+,22-,25-,26-,27+,28+,29-,30+,31-,32+,33+,34+,35+,36-,37-,38-,39+,40-,41+,42+,43-,44+,45+,46-/m1/s1<br>SMILES:<br>CC1CCC2(C(C3C(O2)CC4C3(CCC5C4CC=C6C5(C(CC(C6)OC(=O)C)OC7C(C(C(C(O7)C)

	O)OC8C(C(C(CO8)O)O)O)OC9C(C(C(C(O9)C)O)O)O)C)C)C)OC1
